# Regulation of carboxylesterase 1 (CES1) in human adipose tissue

**DOI:** 10.1016/j.bbrc.2009.03.120

**Published:** 2009-05-22

**Authors:** Margareta Jernås, Bob Olsson, Peter Arner, Peter Jacobson, Lars Sjöström, Andrew Walley, Philippe Froguel, Philip G. McTernan, Johan Hoffstedt, Lena M.S. Carlsson

**Affiliations:** a Department of Molecular and Clinical Medicine and Center for Cardiovascular and Metabolic Research, The Sahlgrenska Academy, 41345 Göteborg, Sweden; b Department of Cell and Molecular Biology, Karolinska Institute, Stockholm, Sweden; c Section of Genomic Medicine, Hammersmith Hospital, Imperial College, London, UK; d CNRS 8090-Institute of Biology, Pasteur Institute, Lille, France; e Diabetes & Metabolism Unit, University of Warwick, Walsgrave, Coventry, UK

**Keywords:** Adipose tissue, Obesity, Human, Carboxylesterase 1, Microarray, Lipolysis

## Abstract

Carboxylesterase 1 (CES1) has recently been suggested to play a role in lipolysis. Our aim was to study the regulation of CES1 expression in human adipose tissue. In the SOS Sib Pair Study, CES1 expression was higher in obese compared with lean sisters (*n* = 78 pairs, *P *= 8.7 × 10^−18^) and brothers (*n* = 12 pairs, *P *= 0.048). CES1 expression was higher in subcutaneous compared with omental adipose tissue in lean (*P *= 0.027) and obese subjects (*P *= 0.00036), and reduced during diet-induced weight loss (*n* = 24, weeks 8, 16, and 18 compared to baseline, *P *< 0.0001 for all time points). CES1 expression was higher in isolated adipocytes compared with intact adipose tissue (*P *= 0.0018) and higher in large compared with small adipocytes (*P *= 4.1 × 10^−6^). Basal and stimulated lipolysis was not different in individuals with high, intermediate, and low expression of CES1. Thus, CES1 expression was linked to body fat and adipocyte fat content but not to lipolytic activity.

## Introduction

Obesity is associated with the development of metabolic syndrome, a major risk factor for cardiovascular morbidity and mortality [1]. Obesity treatment improves metabolic function [2] and reduces overall mortality [3]. The negative energy balance during a hypocaloric diet is known to profoundly modify adipocyte metabolism, particularly the lipolytic pathway [4]. Lipolysis, the process leading to breakdown of triglycerides into fatty acids and glycerol, requires the activities of TG lipases, mainly hormone-sensitive lipase (HSL) and adipose triglyceride lipase (ATGL) [5].

The mammalian CES genes are members of the serine esterase family and the encoded proteins are localized in the endoplasmic reticulum (ER) of many tissues. These enzymes efficiently catalyze the hydrolysis of a variety of ester- and amide-containing chemicals [6]. In the mouse, carboxylesterase 3 (CES3; also called triacylglycerol hydrolase; TGH), the homolog of human CES1, is responsible for a major fraction of adipose tissue lipolytic activity *in vitro* [7], and it has also been suggested that CES1 is a TG lipase in man [8], however evidence is lacking.

The aim of this study was to examine CES1 expression in human adipose tissue in relation to obesity, weight loss, adipocyte size, adipose tissue location, and lipolysis.

## Materials and methods

All study subjects received written and oral information before giving written informed consent. The regional Ethics Committee in Gothenburg, at the Karolinska University Hospital and the South Birmingham Ethics Committee approved these studies. Blood samples and adipose tissue biopsies were taken after an overnight fast. Biochemical and anthropometrical measurements and examinations were performed as described [9,10]. Computerized tomography (CT) was used to determine body composition as previously described [11].

### Subjects and samples

*Very low calorie diet (VLCD) studies*. Twenty-four unrelated obese subjects, 6 women and 18 men, were given VLCD (450 kcal/day) for 16 weeks followed by gradual reintroduction of a normal diet. Abdominal subcutaneous adipose tissue biopsies and fasting blood samples were obtained before (week 0), during (weeks 8 and 16), and after (week 18) treatment. On average, the patients lost 27.7 kg during the diet [12]. Microarray analysis was performed individually in all 24 subjects at all time points. For verification by real-time PCR, abdominal subcutaneous adipose tissue biopsies were obtained from 10 additional subjects at baseline, after 8 and 18 weeks of VLCD.

*The SOS Sib Pair Study*. The SOS Sib Pair Study consists of 154 nuclear families with BMI-discordant sib pairs (BMI difference ⩾ 10 kg/m^2^) resulting in a study population consisting of 732 subjects. Microarray expression analysis was performed in the offspring cohort. Normalization was performed using MAS 5.0 (Affymetrix, Santa Clara, CA). For correlations, results from 357 microarrays were included. However, for comparisons between lean and obese siblings, gender discordant pairs were excluded and only the most BMI-discordant sib pair in each family was included, resulting in 78 pairs of sisters and 12 pairs of brothers.

*Paired abdominal subcutaneous and omental adipose tissue*. Paired abdominal subcutaneous and omental (*n* = 10) adipose tissue was collected from female patients (age 45.4 ± 9.2 years; mean ± SD) undergoing elective surgery (liposuction and elective gynecological procedures), in accordance with guidelines of the South Birmingham Ethics Committee. Smokers and subjects with recent weight change, hormone replacement, and malignant diseases were excluded. The subjects were divided into two groups according to BMI (lean, BMI 23.0 ± 1.2 kg/m^2^; obese, BMI 33.2 ± 3.1 kg/m^2^; mean ± SE). Microarray analysis was performed individually on samples from all 10 subjects.

*Large and small adipocytes*. Needle biopsies of subcutaneous abdominal adipose tissue were obtained from 3 (microarray analysis) and 5 healthy volunteers (real-time PCR analysis), and small and large adipocytes were isolated as previously described [13].

*Isolated adipocytes and intact adipose tissue*. Subcutaneous abdominal adipose tissue biopsies were obtained by needle aspirations in 14 subjects. A fraction was immediately frozen at −70 °C, and from the remaining adipose tissue, adipocytes were isolated by collagenase digestion [14]. RNA was prepared and real-time PCR analysis was performed on isolated adipocytes and intact adipose tissue.

*Studies of lipolysis*. Subcutaneous abdominal adipose tissue biopsies were obtained by needle aspirations in 81 obese women under local anesthesia (BMI 31–53 kg/m^2^ and age 21–63 years). A fraction (300 mg) was immediately frozen at −70 °C and used for subsequent mRNA analysis, and the remaining tissue was used for lipolysis studies. The lipolysis assay has previously been described in detail [15]. In brief, a diluted suspension of isolated fat cells (about 5000–10,000 cells/ml) was incubated for 2 h in duplicate with air as the gas phase at 37 °C in Krebs–Ringer phosphate buffer (pH 7.4) supplemented with glucose (8.6 μmol/ml), ascorbic acid (0.1 mg/ml), and bovine serum albumin (20 mg/ml) in the absence (basal) or presence of increasing concentrations noradrenaline (a non-selective β- and α_2_-adrenoreceptor agonist) or insulin. 8-Bromo-cyclic AMP (1 mmol/l, phosphodiesterase non-resistant cyclic AMP analogue) was added in the insulin experiments in order to magnify the antilipolytic effect of insulin. After the incubation, glycerol was measured in the media (lipolysis index) using a bioluminescence method [15]. The rate of lipolysis was expressed as μmol glycerol per 10^7^ cells per 2 h. Basal and 8-bromo-cyclic AMP-affected glycerol release and the glycerol release at the maximum effective concentration for noradrenaline and insulin were calculated.

### RNA preparation and DNA microarray analysis

Total RNA from adipose tissue was prepared with Qiagen Lipid tissue kit (Qiagen, Hilden, Germany). Total RNA from adipocytes was prepared with the phenol–chloroform extraction method of Chomczynski and Sacchi [16]. After further purification with RNeasy (Qiagen), the RNA concentration was measured spectrophotometrically. The *A*_260_/*A*_280_ ratio was 1.8–2.0 and the quality of the RNA was verified by agarose gel electrophoresis before reverse transcription into cDNA. Gene expression was measured using the Human Genome U133A or U133Plus2.0 DNA microarray (Affymetrix). Preparation of cRNA and hybridization was performed according to standard Affymetrix protocols as previously described [9].

### Real-time PCR analysis of gene expression

Reagents for real-time PCR analysis of CES1 and the reference genes LDL receptor-related protein 10 (LRP10) and 18S rRNA (Assays-on-Demand, TaqMan Reverse Transcriptase reagents, and TaqMan Universal PCR Master Mix) were from Applied Biosystems (Foster City, CA) and used according to the manufacturer’s protocol. cDNA corresponding to 10 ng of RNA per reaction was used for real-time PCR analysis. Specific products were amplified and detected with the ABI Prism 7900HT Sequence Detection System (Applied Biosystems) using default cycle parameters. A standard curve obtained by serial dilution of pooled adipocyte cDNA was plotted for each primer–probe set. All standards and samples were analyzed in triplicate.

### Statistical analysis

Correlations between CES1 expression and anthropometric and biochemical markers were performed using the Spearman rank correlation test. Differences in gene expression between cell populations were analyzed using the Wilcoxon signed-rank test. Differences in CES1 expression between lean and obese siblings were assessed using a paired *T*-test. Correlation of within person longitudinal measurements for the overall relations across the entire VLCD study duration was addressed through the use of generalized estimating equations [17], as previously described [12]. Values are expressed as mean ± SD.

## Results

### Diet-induced weight loss

**Fig. 1:**
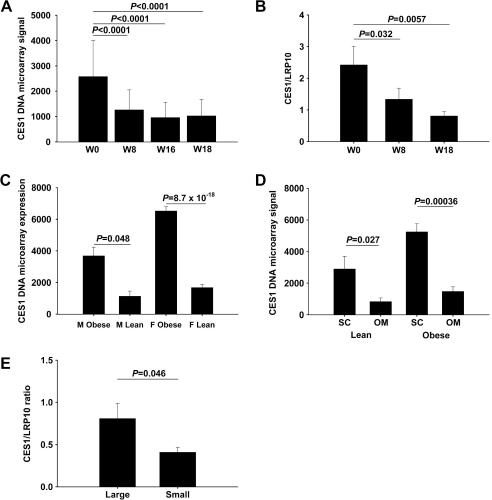
Regulation of CES1 expression in human adipose tissue. (A) Adipose tissue CES1 expression during diet-induced weight loss measured by DNA microarray analysis (*n* = 24). (B) Adipose tissue CES1 expression during diet-induced weight loss measured by real-time PCR (*n* = 10). (C) CES1 expression in adipose tissue of obese and lean siblings (12 pairs of brothers and 78 pairs of sisters) measured by DNA microarray analysis. (D) CES1 expression in paired abdominal and omental adipose tissue samples from 5 obese and 5 lean women measured by DNA microarray analysis. (E) CES1 expression in isolated small and large adipocytes measured by real-time PCR (*n* = 5).

DNA microarray analysis showed that CES1 expression was reduced in subcutaneous adipose tissue after 8 and 16 weeks of VLCD compared with baseline (*P *< 0.0001 and *P *< 0.0001, respectively, Fig. 1A). CES1 expression remained significantly reduced compared with baseline also after the 2 weeks of reintroduction to normal food (week 18; *P *< 0.0001; Fig. 1A). The reduced CES1 expression at weeks 8 and 18 compared to baseline was verified by real-time PCR in another 10 subjects treated with VLCD (*P *= 0.032 and *P *= 0.0057, respectively, Fig. 1B).

CES1 expression levels in the VLCD study correlated with several anthropometric and biochemical parameters, including total adipose tissue, subcutaneous adipose tissue, OGTT insulin, cholesterol, low density lipoprotein cholesterol (LDL-C), and high density lipoprotein cholesterol (HDL-C) (Table 1).

### Obesity-discordant siblings

In the SOS Sib Pair Study, obese females and males displayed higher CES1 expression compared with their lean siblings of the same gender (females *P *= 8.7 × 10^−18^ and males *P *= 0.048; Fig. 1C). CES1 expression levels correlated with several anthropometric and biochemical parameters, including BMI, waist, waist hip ratio, systolic blood pressure, diastolic blood pressure, OGTT, F-insulin, HOMA, cholesterol, LDL-C, HDL-C, and hs-CRP (Table 2).

### CES1 expression in adipose tissue depots and adipocytes

CES1 expression was higher in isolated adipocytes compared with intact adipose tissue (*P* = 0.0018) and higher in subcutaneous compared with omental adipose tissue in both obese and lean subjects (obese, *P* = 0.00036; lean, *P* = 0.027; Fig. 1D). Moreover, CES1 expression was higher in large compared with small adipocytes from the same biopsy measured by DNA microarray (*P* = 4.1 × 10^−6^), and this was verified with real-time PCR (*P* = 0.046, Fig. 1E).

### CES1 expression in relation to lipolysis

**Fig. 2:**
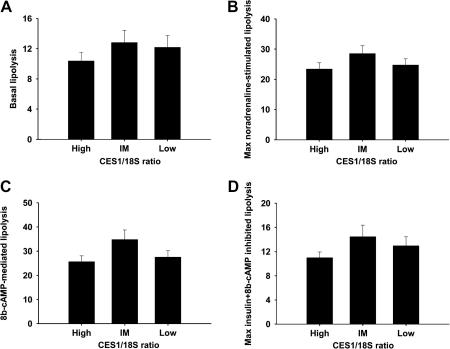
Relation between CES1 expression and lipolysis in isolated adipocytes. Basal lipolysis (A), maximal noradrenaline-stimulated lipolysis (B), 8b-cAMP-mediated lipolysis (C), and 8b-cAMP + insulin-inhibited lipolysis (D) in samples with high, intermediate, and low CES1 mRNA levels measured by real-time PCR.

To examine the relation between CES1 expression and lipolysis, adipocytes from different individuals were divided into tertiles based on their relative CES1 expression measured by real-time PCR. There were no differences in either basal or 8b-cAMP-mediated lipolysis between samples with low, intermediate, and high CES1 expression. Moreover, both noradrenaline-stimulated and insulin-inhibited lipolysis was similar in samples with high, intermediate, and low expression levels of CES1 mRNA (Fig. 2A–D).

## Discussion

In this study, we investigated the regulation of CES1 expression in human adipose tissue and the relation between CES1 expression and TG lipolysis in adipocytes. Our results show that the expression of the CES1 gene is highly regulated in human adipose tissue, with increased levels in obese subjects and decreased levels during weight loss. However, there was no association between CES1 expression and lipolysis in isolated human adipocytes.

In our study, CES1 expression was markedly higher in adipose tissue from obese compared to lean subjects, which is in accordance with the results reported by Steinberg et al. [8]. In obese subjects, diet-induced weight loss resulted in decreased CES1 expression. During the VLCD study, CES1 expression correlated with total and subcutaneous adipose tissue mass measured by CT. CES expression was higher in adipocytes compared to whole adipose tissue, suggesting that the adipocyte is the main site of CES1 expression in adipose tissue. In addition, large adipocytes have higher CES1 expression compared with small adipocytes. Taken together, our results indicate that adipose tissue expression of CES1 is tightly linked to obesity, amount of body fat, and adipocyte fat content.

It has been suggested that CES3, the mouse homolog of human CES1, plays a major role in stimulated and basal lipolysis in mice [7]. To clarify the role of CES1 in human adipose tissue we analyzed basal, stimulated and inhibited lipolysis in isolated human adipocytes and found no differences in samples with low, intermediate, and high CES1 expression. Since differences in CES1 expression were not accompanied by differences in lipolytic activity our data do not provide further support for the idea that CES1 is an important lipase in human adipose tissue. Schweiger et al. recently demonstrated that 95% of the lipolysis in adipose tissue in mice is mediated via ATGL and HSL [18], thereby also questioning the importance of other lipases such as CES1. Studies on the main lipases, HSL and ATGL, show that their regulation in human adipose tissue differs from the regulation of CES1. HSL expression is reduced in obesity [19–22] and the expression is increased after weight reduction [19]. ATGL expression on the other hand is not different in adipose tissue from obese compared to lean subjects and it is not affected by weight reduction [19]. In both the VLCD study and in the Sib Pair Study, CES1 expression in adipose tissue correlated with metabolic parameters, including total cholesterol, LDL-C, and HDL-C. Interestingly, it has been suggested that CES1, addition to its proposed lipolytic activities, also affects a variety of endogenous cholesterol metabolism pathways, including cholesterol esterification and hydrolyzation of cholesterol esters [23,24]. However, further studies are needed to determine if the associations between CES1 expression and plasma lipid levels are linked to effects of CES1 on cholesterol metabolism.

We conclude that the expression of CES1 is associated with body fat and adipocyte fat content. However, CES1 expression was not associated with the lipolytic activity in isolated adipocytes. Taken together, our data show that increased CES1 expression is associated with obesity and markers of metabolic dysfunction indicating that CES1 plays a role in human metabolism, however, the mechanisms are unclear.

*Disclosure statement:* M.J., B.O, P.A, P.J, L.S, A.W, P.F, P.G.M, J.H., and L.M.S.C. have nothing to declare.

## Figures and Tables

**Table 1:** Characteristics of the patients included in the VLCT study and association of CES1 expression, analyzed by DNA microarray, with anthropometric measurements and metabolic parameters during diet-induced weight loss.

					CES1	
	Week 0	Week 8	Week 16	Week 18	Slope	*P*-value
BMI (kg/m^2^)	37.6 ± 4.9	31.8 ± 4.1	28.6 ± 4.1	28.9 ± 3.9	0.067	0.30
Waist (cm)	123 ± 12	110 ± 12	101 ± 13	101 ± 13	0.051	0.51
WHR	1.02 ± 0.08	0.99 ± 0.08	0.95 ± 0.08	0.95 ± 0.08	0.26	0.060
TAT (cm^2^)	778 ± 191		419 ± 174		0.056	0.016
SAT (cm^2^)	526 ± 166		311 ± 137		0.057	0.0062
VAT (cm^2^)	241 ± 76		101 ± 49		0.14	0.46
S-BP (mmHg)	138 ± 17	121 ± 12	117 ± 14	124 ± 16	0.056	0.44
D-BP (mmHg)	88 ± 13	76 ± 11	72 ± 11	76 ± 12	−0.089	0.063
F-glucose (mmol/L)	6.0 ± 1.6	4.5 ± 0.7	4.5 ± 0.7	5.0 ± 1.0	0.51	0.26
OGTT glucose (mmol/L)	8.2 ± 3.8	7.0 ± 1.9	7.0 ± 2.6	5.9 ± 2.3	0.58	0.056
F-insulin (mU/L)	15.8 ± 7.4	7.0 ± 4.1	4.3 ± 2.2	6.3 ± 3.7	0.37	0.081
OGTT insulin (mU/L)	67 ± 43	41 ± 21	34 ± 17	24 ± 10	0.46	0.0051
HOMA	4.4 ± 2.7	1.4 ± 0.9	0.9 ± 0.5	1.5 ± 1.3	0.31	0.066
Total C (mmol/L)	5.6 ± 0.2	3.9 ± 0.1	4.4 ± 0.1	4.8 ± 0.1	2.05	0.0054
LDL-C (mmol/L)	3.6 ± 1.0	2.3 ± 0.8	2.6 ± 0.6	2.9 ± 0.7	0.068	0.041
HDL-C (mmol/L)	1.4 ± 0.4	1.2 ± 0.3	1.4 ± 0.4	1.4 ± 0.3	1.28	0.046
TG (mmol/L)	1.8 ± 1.0	1.0 ± 0.2	0.9 ± 0.2	1.2 ± 0.5	0.21	0.53
hs-CRP (mg/L)	5.3 ± 5.8	4.6 ± 5.4	2.4 ± 1.5	2.4 ± 2.2	−0.10	0.53
Leptin (ng/L)	35.6 ± 3.2	8.6 ± 1.2	5.3 ± 0.9	7.6 ± 1.1	0.047	<0.0001
Adiponectin (μg/ml)	8.2 ± 0.8	10.4 ± 1.1	12.5 ± 1.05	14.1 ± 1.3	0.50	0.12

BMI, body mass index; WHR; waist hip ratio; TAT, total adipose tissue; SAT, subcutaneous adipose tissue; VAT, visceral adipose tissue; S-BP, systolic blood pressure; D-BP, diastolic blood pressure; F-glucose, fasting plasma glucose; C, cholesterol; LDL, low density lipoprotein; HD, high density lipoprotein; TG, triglyceride; hs-CRP, high sensitive C-reactive protein, *n* = 24. The clinical characteristics of subjects in the VLCD study have been described before [10,12,25,26].

**Table 2:** Correlations between adipose tissue CES1 expression and anthropometric and biochemical parameters in the SOS Sib Pair Study.

		CES1	
		*P*-value	*r*
BMI (kg/m^2^)	29.6 ± 8.2	<0.0001	0.66
Waist (cm)	95.9 ± 19.3	<0.0001	0.63
WHR	0.9 ± 0.09	<0.0001	0.45
S-BP (mmHg)	118 ± 15	<0.0001	0.22
D-BP (mmHg)	72 ± 11	<0.0001	0.24
F-glucose (mmol/L)	5.0 ± 1.1	0.28	0.057
F-insulin (mU/L)	9.6 ± 7.6	<0.0001	0.54
HOMA	2.2 ± 2.1	<0.0001	0.51
Total-C (mmol/L)	4.4 ± 0.9	0.0446	0.077
LDL-C (mmol/L)	2.7 ± 0.7	<0.0001	0.18
HDL-C (mmol/L)	1.2 ± 0.3	<0.0001	−0.38
TG (mmol/L)	1.1 ± 0.7	<0.0001	0.37
hs-CRP (mg/L)	4.3 ± 6.3	<0.0001	0.50

BMI, body mass index; WHR, waist hip ratio; S-BP, systolic blood pressure; D-BP, diastolic blood pressure; F-glucose, fasting plasma glucose; C, cholesterol; LDL, low density lipoprotein; HDL, high density lipoprotein; TG, triglyceride; hs-CRP, high sensitive C-reactive protein, *n* = 357.
